# A Mixed-Methods Sequential Explanatory Study of the Factors That Impact Nurses’ Perspectives toward Nurse Practitioners’ Roles in Saudi Arabia

**DOI:** 10.3390/healthcare11010146

**Published:** 2023-01-03

**Authors:** Ali Kerari, Ghareeb Bahari, Nahed Aldossery, Omaimah Qadhi, Alya Alghamdi

**Affiliations:** 1 Medical Surgical Department, College of Nursing, King Saud University, Riyadh 11421, Saudi Arabia; 2 Nursing Administration and Education Department, College of Nursing, King Saud University, Riyadh 11421, Saudi Arabia; 3 Nursing Specialist in Riyadh, Riyadh 12511, Saudi Arabia; 4 Community, Psychiatric and Mental Health Nursing Department, College of Nursing, King Saud University, Riyadh 11421, Saudi Arabia

**Keywords:** nurse practitioners, perspectives, nursing, roles

## Abstract

Nurse practitioners’ roles need to be clearly defined in Saudi Arabia. Therefore, we aimed to explore potential factors that impact nurses’ perspectives toward nurse practitioners’ roles and whether they are interested in becoming nurse practitioners. A mixed-methods study design was employed using a questionnaire and focus groups. The survey was sent to nurses working at a public hospital in the Riyadh region. Participants (N = 77) reported that having more nurse practitioners would improve quality of care and patient safety. Additionally, most participants stated that an increased supply of nurse practitioners would have a positive impact on effectiveness, equity of care, and healthcare costs. In regression analysis, participants with favorable perspectives towards nurse practitioners were significantly more likely to have interest in becoming nurse practitioners (odds ratio [95% confidence interval]:1.04 [1.01–1.07]). In the qualitative domain, three positive factors were identified: effective collaboration with other staff, better contribution to quality care and patient safety, and better contribution to evidence-based practice. Two barriers were also determined: lack of motivation to become a nurse practitioner and unclear scope of practice. Results showed that nurse practitioners can provide quality healthcare services that meet patients’ different needs. The growing role of this speciality warrants further research to show its value in daily practice.

## 1. Introduction

The job titles used in advanced nursing positions vary worldwide and often include nurse practitioner, nurse clinician, non-physician, and advanced practice nurse [1]. In Saudi Arabia, the Ministry of Health distinguishes nurses’ roles based on their education level [2]. Nurse practitioners are prepared with advanced clinical knowledge, education, skills, and decision-making abilities in complex situations [3]. They are highly qualified and competent healthcare professionals who can act in many settings [4], including primary care and pre- and post-operative care units to provide cost-effective, accessible, patient-centered medical care. As mentioned in the literature [2], the International Council of Nurses defines nurse practitioner as:

“A registered nurse who has acquired the expert knowledge base, complex decision-making skills and clinical competencies for expanded practice, the characteristics of which are shaped by the context and/or country in which s/he is credentialed to practice. A master’s degree is recommended for entry level.”

(p. 72)

Evidence suggests that nurse practitioners can provide higher levels of care, achieve successful outcomes, and help with the shortages of primary care physicians [5]. They may even achieve better outcomes in terms of improving patient satisfaction, reducing wait times, and managing chronic diseases [6]. Increasing the supply of nurse practitioners would also result in greater collaboration with physicians [7]. Nurse practitioners are known to perform a wide range of functions, such as assisting patients with decision making, fostering engagement between patients and their families, delivering continuity of care, enhancing patient satisfaction, advancing interpersonal communication and professional relationships, reducing morbidity and mortality, improving treatment compliance and productivity, and minimizing healthcare costs [8]. When comparing nurse practitioners with other nurses, researchers conducting a study in Australia reported that nurses in the field of advanced practice demonstrate patterns of practice different from other staff nurses [9]. Further, nurse practitioners are permitted to prescribe treatments, order tests, and complete patient diagnoses. Such duties are usually performed by physicians.

The nurse practitioner specialty is an important part of the workforce, with the potential to transform and improve care across cultures [10]. The World Health Organization raised a concern about the Saudi healthcare system, encouraging Saudi Arabia to implement radical changes to the function and structure of nursing for high-quality and effective delivery of care [11]. Implementing the Saudi Healthcare Transformational Plan would facilitate this change, providing evidence for the provision of resources and a foundation for using nurse practitioners in Saudi Arabia [12].

Recently, addressing the need for nurse practitioners in the country by discussing barriers and enablers to implementation has become a main focus [2]. Four pillars which were necessary to formalize nurse practitioners at the national level were proposed: legislation, regulation, credentialing, and privileging. There was emphsasis was on the importance of assigning a national committee of stakeholders, formulating legislation, recognizing the successful Saudi nurse practitioner models, investigating the gaps in the healthcare system, providing the necessary training and coaching, and promoting qualified nurse practitioners [2]. Formalizing the nurse practitioners’ role is a critical step toward addressing the challenges and improving their engagement in clinical practice [13].

Nurse practitioner speciality brings comprehensive perspectives and advanced skills in practice. Although such speciality has received significant attention, we believe there is a need for appropriate legislation that lays out clear policies and procedures for its roles. Furthermore, the field of nursing is facing shortages across the country, which limits the growth and tranformations of the profession. There is, therefore, a need to improve the practice of nursing and make it more appealing [2]. Further, there may be more challenges associated with nursing advancement in the country. It is important to explore the perspectives of the nurses in this profession and their intentions to become nurse practitioners. Thus, addressing the gaps in the literature and in clinical practice, the purpose of this study was to determine potential factors that impact nurses’ perspectives toward nurse practitioners’ roles in Saudi Arabia. Three main questions were addressed: (1) What are nurses’ perspectives toward nurse practitioners’ roles in Saudi Arabia? (2) How many nurses are interested in becoming nurse practitioners? (3) What are potenial factors and barriers for the nurse practitioner specialty in Saudi Arabia?

## 2. Materials and Methods

### 2.1. Design and Settings

This study examined nurses’ perspectives of nurse practitioners’ roles in Saudi Arabia through a mixed-methods approach within a sequential explanatory design [14]. The study was conducted in two parts, the first employing a quantitative cross-sectional design, in which data were collected from many different participants at a single point in time [15]. The second stage involved gathering qualitative descriptions of nurses’ views of nurse practitioners’ roles. The study was conducted at a public hospital with approximately 1500 beds, 200 of which serve patients admitted to the surgical department. There are around 1400 nurses working there in total (A., Al-Asmari, personal communication, 2 January 2022). The hospital is located in the Riyadh region of Saudi Arabia.

### 2.2. Sampling Process

A convenience sample of 77 nurses was selected as a sample size and was determined using Morgan’s table [16]. Both Saudi and non-Saudi registered nurses that had been working at the hospital for at least one year and had an understanding of the nurse practitioners’ role were able to participate in the study. Nursing students who had passed the licensure exam but were still in their internship year were excluded because they likely had limited knowledge of the nurse practitioners’ scope of practice. In addition, nurses with low English proficiencies were excluded, as they would likely be unable to understand the questionnaire items.

For the qualitative part of the study, a purposive sample strategy was used to recruit participants. Nurses who were interested in participating in the focus group session and had agreed to be recorded were able to participate. We also aimed to include nurses who did not participate in the questionnaire, as they could provide in-depth and diverse knowledge regarding nurse particioners’ roles. Further, though English is the official language of nursing in Saudi Arabia, we met only with Arabic speakers in order to collect as much information as possible. Sample size was determined by data saturation, meaning that no additional details were being found or provided by participants. Next, the research team agreed on the adequacy of the information collected.

### 2.3. Data Collection Procedures

#### 2.3.1. Quantitative Phase

Quantitative data were collected from March 2022 to June 2022 using an anonymous online questionnaire. The questionnaire items assessed demographic information and perspectives toward nurse practitioners’ roles. The demographic survey included specific individual factors (i.e., age, gender, nationality, level of education, work status, department type, years of experience, and previous experience with nurse practitioners). The instrument used to measure nurses’ perspectives included 34 items from three different sections [1]. The first 13 questions focused on nurses’ opinions and perspectives on nurse practitioners’ scope of practice and the need for practitioners in the nursing field. These questions were adapted from the “US National Survey of Primary Care Nurse Practitioners and Physician” to improve clarity [1]. The next 17 items examined thoughts on the role and professional image of nurse practitioners. They were developed according to Strong’s model for advanced practice nursing [1]. The last four items targeted current nurses’ interests in becoming nurse practitioners. The instrument’s face validity was ensured by five nurse practitioner experts, an educator, a Ministry of Health administrator, and an advanced practice nursing researcher; they indicated an agreement of the final instrument [1]. The Cronbach’s alpha of the scale in this study was determined to be 0.94. Nurses were emailed the study link by unit managers. A research assistant also helped with distributing the questionnaire link to nurses.

#### 2.3.2. Qualitative Phase

An announcement of the qualitative part of the study was posted on Twitter, the most-used social media site in Saudi Arabia. Interested nurses contacted the research team members indicating their interest to participate in the study. Due to participants’ busy work schedules, two focus group sessions were arranged. Approximately ten registered nurses who met the inclusion criteria completed the two sessions, which were conducted virtually using Zoom. Zoom is a reliable program that is used to facilitate virtual communication and arrange meetings and events. Before starting each session, participants were assured that their information and responses were kept confidential and accessible only to the research team members.

Each focus group session lasted for approximately 60 min. The focus group sessions were conducted in Arabic to ensure that participants could easily and clearly express their opinions. Each was then translated into English by an individual who was bilingual and familiar with the nursing system in Saudi Arabia. Participants were asked about their views on some of the results obtained in the quantitative research. Other questions were specifically about the role of colleges in teaching the nurse practitioner specialty. Additional questions targeted strengths and weaknesses of nurse practitioners at hospitals and whether they contribute to better quality of care and patient safety. Questions based on participants’ views (e.g., “why?”, “how?” or “what do you mean by this?”) were asked to elicit more useful details. Data saturation was confirmed based on nurses’ responses. The quality of qualitative data was ensured by the following criteria: credibility, transferability, dependability, and confirmability [17].

### 2.4. Ethical Considerations

The study was approved by the institutional review board of King Saud Medical City (Reference #: H1RI-27-Jan22-01). Participation was completely anonymous and voluntary, and informed consent was ensured prior to data collection. All nurses who participated in the qualitative interview agreed to be audio recorded. For confidentiality purposes, a code (RN) was used to refer to participants when reporting findings. All data relating to the study will be retained and available for at least 3 years after the study end date. After that, all responses and transcripts will be destroyed.

### 2.5. Data Analysis

Collected data were analyzed using SPSS (v.28) (IBM Corp., Armonk, NY, USA). Frequency distributions were run to identify missing data, outliers, or data entry errors. No more than 2% of data were missing for any item, and missing data were replaced by the item mean [18]. Logistic regression was also run to evaluate nurses’ perspectives toward nurse practitioners and covariates on their interests in becoming nurse practitioners.

Both focus groups were audio recorded and transcribed verbatim. A thematic analysis method was used to search for themes in the data. The second author (G.B.) listened to the records and read the transcripts several times to confirm full understanding of participants’ answers. All responses were then organized in a Microsoft Word document to help highlight similar responses between participants. Initial data coding was generated by the third author, then reviewed and confirmed by other team members. The final codes were grouped, and the relationships between them were identified. When themes were clear in the final codes, they were reviewed, discussed with the team members, and edited as necessary. Quoted sentences were added to support each of them. Similar processes have been performed in prior studies [19,20]. A qualitative expert was also asked to review and confirm the analysis.

## 3. Results

### 3.1. Quantative Results

#### 3.1.1. Sample Characteristics

Table 1 provides the sample characteristics of the study (N = 77). Participants’ mean age was 33.44 years (SD ± 4.94 years; range = 24–45 years). Most participants were female (89.6%), Saudi (90.9%), and had been working in the field of nursing for approximately five to ten years (44.2%). The majority of participants held a college degree in nursing (63.7%) and received more than SR 7000 (87%) (equivalent to USD 1866) monthly. Further, about 61% of participants were on a permanent contract and were accustomed to working with nurse practitioners. Approximately one third of the sample worked in critical and surgical units (31.2%). However, about 18.2% of participants reported the training duration as a main constraint that prevented them from pursuing the nurse practitioner specialty. Almost 56% of the sample were identified by unit managers as suitable candidates for the nurse practitioner role. Most participants (78%) also indicated an interest in becoming a nurse practitioner.

#### 3.1.2. Supply of Nurse Practitioners

We asked the study participants to consider whether increasing the supply of nurse practitioners in Saudi Arabia would improve, worsen, or have no effect on multiple aspects of care quality (see Table 2). The majority of participants reported that having more nurse practitioners would improve quality of care (79.2%) and safety (80.5%). Additionally, most participants stated that an increased supply of nurse practitioners would have a positive impact on efficacy, equity of care, and healthcare costs.

#### 3.1.3. Nurse Practitioners’ Roles

A total of 93.5% of the participants believed that nurse practitioners “should practice to the full extent of their education and training”, while 87.1% agreed that nurse practitioners provided as effective healthcare services as physicians. Of the nurses surveyed, 37.7% agreed that nurse practitioners “should be legally allowed hospital-admitting privileges in outpatient settings”. Further, 40.3% also agreed that the cost of outpatient care delivered by nurse practitioners should be comparable to that of care delivered by physicians (see Table 3).

Factors influencing nurse practitioners’ professional image were addressed, including levels of clinical knowledge and expertise, professional and leadership skills, medical decision-making skills, and contributions to the health institution and hierarchical relations. In terms of interprofessional teams, the majority of participants (80.8%) felt that nurse practitioners were actively involved in medical decision-making and had displayed sufficient clinical expertise in and patient care and management. Most nurses also considered nurse practitioners as taking part in leadership (85.5%), education (81.9%), and research (86.8%). In addition, nurses regarded nurse practitioners as key figures in improving the public image of nursing (88.4%) (Table 3).

In summary, respondents’ views were reported on every measure of policies and practices related to the nurse practitioners’ scope of practice. In addition, nurse practitioners’ professional image was evaluated by the perceived roles they played in clinical knowledge and expertise and contributions to the health care system. The findings suggest that participants generally viewed the role and professional image of nurse practitioners positively.

#### 3.1.4. Interest in Becoming Nurse Practitioners

Using a logistic regression, the outcome variable of interest in becoming nurse practitioners was coded as a binary option (Yes = 1, No = 0). Table 4 displays the standardized beta, standard error, Wald, significance, odds ratio, and lower and upper range of the 95% confidence intervals of the logistic regression for interest in becoming nurse practitioners. The logistic regression met the Hosmer—Lemeshow goodness-of-fit test with a Chi-square value of 5.70, seven degrees of freedom, and a significance of 0.58 (*p* > 0.05). The Omnibus Test of Model Coefficient had a Chi-square value of 8.85 and eight degrees of freedom. As shown in Table 4, the logistic regression results revealed that perspectives toward nurse practitioners were predictor variables that were statistically significant contributors to the model (*p* < 0.05). Participants with higher scores on the perspectives towards nurse practitioners subscale were significantly more likely to have interest in becoming nurse practitioners (odds ratio [95% confidence interval]:1.04 [1.01–1.07]). The remaining covariates (e.g., age, gender, education, income, and years of experience) were not significant predictors of interest in becoming a nurse practitioner.

### 3.2. Qualitative Results

A total of eight female and two male nurses participated in the qualitative part of this study. The majority of them were married. When transcript analysis was completed, three positive factors and two barriers to becoming a nurse practitioner had emerged:

#### 3.2.1. Factor One: Effective Collaboration with Other Staff

All participants believed that nurse practitioners would help enhance healthcare services provided to patients. They also believed that collaboration between healthcare staff would be improved with the presence of nurse practitioners. Example comments include:

“It may be easy to work with nurse practitioners because current nurses have the experience, while nurse practitioners have the information. There would be effective cooperation between experience and information.”

(N11)

“There was effective cooperation between nurses who hold a diploma and those who have a bachelor’s degree during the COVID-19 period, especially in the ICU. The same idea will surely be with those who have practitioner level.”

(N6)

#### 3.2.2. Factor Two: Better Contribution to Care Quality and Patient Safety

Most participants agreed that nurse practitioners with high levels of education and skills can contribute effectively to improve care quality and patient safety. Participants commented:

“In every health field, there will be development and progress as long as there is a nurse practitioner specialty because it combines practice and information and is advanced in its nature at the same time.”

(N7)

“Nurse practitioners may contribute to changing some of the current ideas and to be leaders. They also have a better and faster intervention and may shorten the time with medical intervention. Yes, they have an important role in improving quality and safety.”

(N3)

#### 3.2.3. Factor Three: Better Contribution to Evidence-Based Practice

Participants stated that nurse practitioners’ level of education, readiness to work in different departments, and potential lengthy experience in the workplace may help them to participate in research and contribute to improved evidence-based practice at hospitals. Nurse practitioners could also play a role in improving evidence-based practice at hospitals. Participants commented:

“Nurse practitioner specialization will have a big role—in my opinion—in improving research in the future.”

(N7)

“Nurse practitioners will certainly have a role by virtue of their degree level. Their information will be more deep. They will certainly have a stronger viewpoint, discover errors, and perhaps modify them and develop the workplace.”

(N2)

#### 3.2.4. Barrier One: Low Motivation to Become a Nurse Practitioner

Many participants stated that though they would like to have more practitioners at the hospital, low motivation for becoming one remains a challenge. Example comments include:

“There are not enough stimuli, meaning that the nurse performs several roles or tasks. Some aspects of the job are similar to the doctor’s work and can also work in inpatient or outpatient. There is supposed to be a plan to provide rewarding incentives.”

(N1)

“I notice maybe three quarters of the hospital staff don’t know what a nurse practitioner is. Neither diploma nor bachelor’s holders know this specialty. Sometimes if I say I have a nurse practitioner degree, the response comes: What is this specialty?”

(N8)

#### 3.2.5. Barrier Two: Unclear Scope of Practice

The majority of participants agreed that the scope of nurse practitioners’ practice is still unclear. Some of them stated that nursing in general suffers because its scope and nature are not clearly defined. Quote examples include:

“I think that the nursing office in the hospital does not recognize the specialty of nurse practitioner yet. They did not give it enough importance like other specialties.”

(N8)

“I think the problem is related to the legislative bodies…meaning that they accepted a specific program, but did not make a future plan for the people who will graduate from this program.”

(N1)

## 4. Discussion

This study employed a mixed-method design to evaluate nurses’ perspectives of nurse practitioners’ roles. The first part involved a quantitative cross-sectional study design, which was followed by the qualitative portion that investigated nurses’ perspectives of nurse practitioners’ roles through focus group discussions.

The results of the quantitative study indicated positive views on the role and professional image of nurse practitioners. Additionally, perspective towards nurse practitioners was the main predictor of participants’ interest in becoming nurse practitioners. Findings of the qualitative study showed that from nurses’ perspectives, effective collaboration with other staff and improved contribution to care quality, patient safety, and evidence-based practice were the most important factors of their interest in becoming nurse practitioners. On the other hand, a lack of motivation and uncertainty about the scope of practice of nurse practitioners were viewed as the most frequent barriers in pursuing the nurse practitioners career track. This paper provides useful information for healthcare legislators and stakeholders when developing a nursing scope of practice and creating a sustainable nursing environment in Saudi Arabia.

Consistent with previous studies [1,21], the results of the quantitative study revealed that nurses generally support an increase of nurse practitioners in Saudi Arabia. The majority of participants reported that quality of care and patient outcomes may improve with more nurse practitioners present in healthcare settings (63–79%). Furthermore, participants considered healthcare provisions by nurse practitioners to be comparable to those by physicians. In line with the results of the quantitative study, the findings of the qualitative study also showed that nurse practice specialization can play an imperative role in advancing healthcare systems and evidence-based practice. Based on past studies [22,23], nurses who participate in continuing education programs, including nurse practitioner programs, are more likely to use evidence-based practice in their daily undertakings. Moreover, 77% of the nurses in this study felt that the increases in knowledge and skills were two of the benefits of becoming nurse practitioners. Considering the nurses’ optimism toward the roles of nurse practitioners, the health workforce needs to be future-ready through role expansion of the nursing profession to promote productivity and effectiveness [24].

In particular, the impact of an expanded workforce of nurse practitioners on healthcare quality needs to be highlighted to develop the Saudi healthcare workforce [2]. Among the respondents to our survey, 93.5% supported the notion that nurse practitioners should practice to the “full extent of their education and training”, the importance of which is emphasized by the American Nurses Association. Similarly, the findings of the quantitative study revealed that nurse practitioners can deliver a wide range of primary healthcare services and actively take part in leadership, education, research, and management. This suggests that the Saudi healthcare system needs to delineate nursing roles, including ANPs, to expand the nursing scope of practice [25].

As the nursing scope of practice advances, the shifting of tasks from physicians to nurses has gained remarkable interest in healthcare policy. However, healthcare systems have been confronted with challenges regarding the implementation and development of nurse practitioners [1]. An undefined scope of practice for nurse practitioners may create confusion among other healthcare professionals. In Saudi Arabia, advanced nursing practice has not been well-implemented, except in a few hospitals (i.e., King Faisal Specialist Hospital and Research Centre) [2]. The qualitative findings showed that unclear scope of practice was an important limitation to becoming a nurse practitioner. In similar studies, other factors that inhibit the pursuit of advanced nursing programs include lack of awareness, motivation, and financial support [26].

On the basis of the logistic regression model, this study investigated the likelihood of interest in becoming a nurse practitioner based on knowledge of nurse practitioners’ roles and characteristics of nurses. Thus, knowledge of nurse practitioners’ roles was the only significant predictor of nurses’ interest in becoming nurse practitioners. In other words, nurses who were aware of specific nurse practitioners’ roles had the greatest likelihood of indicating an interest in becoming nurse practitioners. As proposed previously, the preparation for nurse practitioners needs to be initiated in the Saudi healthcare system. The Saudi Commission for Health Specialties and Ministry of Health have a primary role in expanding the nursing profession [27]. Nursing colleges in Saudi Arabia can also create nurse practitioner programs to recruit registered nurses. Such programs may develop nurses’ clinical skills and qualifications to establish their roles as nurse practitioners.

### 4.1. Study Limitations

There are some limitations worth noting. First, the study was conducted at only one site where most nursing staff likely had limited knowledge of the roles of advanced nurse practitioners. A one-site study may also have limited generalizability of findings beyond the sample. Second, due to the time limitations of some participants, qualitative data were collected through focus groups where more specific details could not be obtained as they may have been in individual interviews. Another limitation of the current study is the small sample size, which is due to the lack of the nurse practitioner function at the study location. Further, the translation of participants’ comments into Arabic was done by only one translator. There was no back-translation to assure the accuracy of the responses, which may have impacted the final interpretation of the results. Despite these limitations, this study emphasized current nurses’ perspectives of the nurse practitioners’ roles and assessed whether they are necessary for a higher quality of care.

### 4.2. Study Implications

The findings of this study will emphasize the importance of the advanced nurse practitioners’ role in Saudi Arabia. Policymakers in the Saudi nursing system should use this study as a guide to establish a clear job description for advanced nurse practitioners. Policies can be formulated to describe the basic requirements for the advanced nursing practice, which may include the minimum number of years in clinical practice. A clear description of the advanced nurse practitioner position may lead more nurses to be interested in becoming practitioners in the future. Therefore, nursing schools in Saudi Arabia should launch practitioners’ programs to meet workplace needs. Furthermore, additional research highlighting potential challenges facing nurse practitioners in Saud Arabia is needed.

## 5. Conclusions

Positive perspectives toward the nurse practitioners’ roles were determined. A large portion of the participants indicated that an increased supply of nurse practitioners in Saudi Arabia would have positive effects on the quality of health care. We believe that these findings contribute to the literature on how practitioner nurses perceive various factors to choose the nursing profession. The practical implications can help practitioners to understand the importance of these motivating factors and improve performance at work. We also suggest legislating and developing a policy that solves nurse practitioners’ potential issues despite the advancements in medical practices. The nurse practitioner specialty is still in its infancy; thus, more work is needed to demonstrate its positive impact on patient safety and quality of care.

## Figures and Tables

**Table 1:** Sample characteristics (N = 77).

Characteristics	N	(%)
Age (Years) M = 33.44, SD ± 4.94, range: 24–45		
Gender		
Male	8	(10.4)
Female	69	(89.6)
Nationality		
Saudi	70	(90.9)
Non-Saudi	7	(9.1)
Years of experience		
Less than 5 years	10	(13.0)
5 to 10 years	34	(44.2)
More than 10 years	33	(42.9)
Level of education		
Diploma	27	(35.1)
Bachelors	23	(29.9)
Higher education	26	(33.8)
Income status		
Less than SR 7000	7	(9.1)
SR 7000 or more	67	(87.0)
Work status		
Permanent	47	(61.0)
Contract	29	(37.7)
Work department		
Medical ward	8	(10.4)
Surgical ward	6	(7.8)
Critical care units	18	(23.4)
Other departments	45	(58.4)
Previous experience with nurse practitioners		
No	30	(39.0)
Yes	47	(61.0)
Interested to becoming a nurse practitioner		
No	17	(22.1)
Yes	60	(77.9)

Note: M = Mean; SD = Standard Deviation.

**Table 2:** Participants’ views regarding the influence of an increased supply of NPs on the healthcare system (N = 77).

	Make Better n (%)	Make Worse n (%)	No Effect n (%)	Don’t Know n (%)
Safety	62 (80.5%)	3 (3.9%)	5 (6.5%)	7 (9.1%)
Timeliness	52 (67.5%)	5 (6.5%)	9 (11.7%)	11 (14.3%)
Effectiveness	61 (79.2%)	5 (6.5%)	4 (5.2%)	7 (9.1%)
Efficiency, cost-effectiveness	53 (68.8%)	7 (9.1%)	5 (6.5%)	12 (15.6%)
Equity	46 (59.7%)	7 (9.1%)	12 (15.6%)	12 (15.6%)
Patient-centeredness	63 (81.8%)	4 (5.2%)	4 (5.2%)	6 (7.8%)
Healthcare cost	49 (63.6%)	7 (9.1%)	9 (11.7%)	12 (15.6%)

**Table 3:** Participants’ views toward nurse practitioners’ roles (N = 77).

	Strongly Agree (%)	Agree (%)	Neither Agree nor Disagree (%)	Disagree (%)	Strongly Disagree (%)	N/A (%)
NPs should practice to the full extent of their education and training	44 (57.1%)	28 (36.4)	4 (5.2%)	0	0	1 (1.3%)
The charges of the outpatient service provided by NPs should not be different from that of a physician	32 (41.6%)	31 (40.3)	8 (10.4)	5 (6.5%)	0	1 (1.3%)
When delivering the same outpatient service as physicians, NPs are able to provide the same quality of service	32 (41.6%)	35 (45.5%)	6 (7.8%)	2 (2.6%)	1 (1.3%)	1 (1.3%)
NPs should be legally allowed hospital-admitting privileges in outpatient settings	38 (49.4%)	29 (37.7%)	8 (10.4)	0	0	2 (2.6%)
NPs are partners in medical decision making (i.e., make decisions with physicians regarding patient management)	31 (40.3%)	35 (45.5%)	5 (6.5%)	1 (1.3%)	1 (1.3%)	3 (3.9%)
NPs provide clinical leadership and consultancy in their specific clinical specialty	31 (40.3%)	35 (45.5%)	8 (10.4%)	0	1 (1.3%)	1 (1.3%)
NPs lead, train, and mentor nurses	35 (45.5%)	29 (37.7%)	9 (11.7%)	1 (1.3%)	1 (1.3%)	2 (2.6%)
NPs promote research/quality improvement project	36 (46.8%)	30 (39%)	7 (9.1%)	1 (1.3%)	1 (1.3%)	2 (2.6%)
NPs promote organization level changes	35 (45.5%)	32 (41.6%)	7 (9.1%)	1 (1.3%)	0	2 (2.6%)
NPs advocate for nurses	35 (45.5%)	31 (40.3%)	8 (10.4%)	1 (1.3%)	0	2 (2.6%)
NPs improve the public image of the nursing profession	34 (44.2%)	34 (44.2%)	3 (3.9%)	1 (1.3%)	2 (2.6%)	3 (3.9%)

Note: NPs: nurse practitioners.

**Table 4:** Logistic regression summary analysis for nurses’ interests in becoming NPs (N = 77).

Variable	*B*	S.E.	Wald	Significance	Odds Ratio	95% CI
Constant	−1.10	2.91	0.14			
Perspectives toward NP Roles	0.03	0.01	5.73	0.017	1.04	1.01–1.07
Age	0.003	0.07	0.003	0.96	1.003	0.88–1.14
Gender	0.18	1.37	0.01	0.89	1.19	0.08–17.5
Years of Experience	−0.21	0.42	0.25	0.61	0.81	0.35–1.85
Education	−0.33	0.44	0.56	0.44	0.72	0.30–1.69
Income	−0.39	1.28	0.09	0.76	0.67	0.05–8.35
Work Status	0.24	0.62	0.14	0.70	1.26	0.37–4.29
Work Department	0.18	0.32	0.31	0.57	1.20	0.63–2.28

## Data Availability

Data are not shared due to privacy and ethical restrictions.
